# Effect of Glazing with Different Materials on the Quality of Tuna During Frozen Storage

**DOI:** 10.3390/foods9020231

**Published:** 2020-02-21

**Authors:** Jinfeng Wang, Wenhui Yu, Jing Xie

**Affiliations:** 1 College of Food Science and Technology, Shanghai Ocean University, Shanghai 201306, China; jfwang@shou.edu.cn (J.W.); strfor@163.com (W.Y.); 2 Shanghai Professional Technology Service Platform on Cold Chain Equipment Performance and Energy Saving Evaluation, Shanghai 201306, China; 3 National Experimental Teaching Demonstration Center for Food Science and Engineering, Shanghai Ocean University, Shanghai 201306, China

**Keywords:** glazed tuna, rosmarinic acid, sodium lactate, antioxidant of bamboo leaf, frozen storage

## Abstract

This study investigated and determined the changes in various qualities of tuna samples that were glazed with rosmarinic acid, a bamboo leaf antioxidant, and sodium lactate and stored at −18 °C for 180 days. The water-holding capacity, cooking loss, color, texture, protein content, and total volatile basic nitrogen (TVB-N) were monitored, to study the effect of tuna glazed with different materials on the quality every 30 days. Low-field nuclear magnetic resonance (LF-NMR) was used to measure the water distribution of tuna in this paper. The results showed that the quality of unglazed tuna decreased significantly after 180 days of frozen storage. During frozen storage, the hardness and a* values of RG (glazed with the rosmarinic acid group), SG (glazed with the sodium lactate group), and CG (glazed with the composite of rosmarinic acid, sodium lactate, and the antioxidant of bamboo leaf) tuna decreased slowly, while the malondialdehyde (MDA) value of AG (glazed with the antioxidant of bamboo leaf group) tuna increased slowly. After 180 days of frozen storage, CG tuna had the highest protein content and the lowest TVB-N value, which may have been due to the synergistic effect of glazing materials. The tuna with CG also had the best freshness and quality after frozen storage. Considering the results, a composite of rosemary (0.3%), sodium lactate (3.4%), and antioxidants of bamboo leaves (0.12%) is the best material for glazing tuna.

## 1. Introduction

Tuna meat is one of the most popular sources for making sushi and sashimi in the world, and it has of high economic and nutritional value [1]. The freshness indicators, including redness, the protein content, the water-holding capacity, and other factors, are critical for determining the tuna’s quality and flavor [2]. Normally, tuna is stored in a frozen state, to minimize bacterial growth and the loss of nutrition. However, protein denaturation and lipid oxidation still occur during prolonged frozen storage, resulting in a deterioration of quality in the tuna, such as discoloration, off-flavors, rancidity, dehydration, and drip loss [3]. 

The glazing of frozen foods has been widely used to prevent oxidization and deterioration during the frozen storage [4]. The ice layer acts as a barrier to control moisture transfer and the oxygen uptake of frozen foods. To improve the effect of the ice coating, several kinds of glazing materials are used, including protein hydrolysate [5], polysaccharides [6], and natural compounds. 

Natural compounds have the advantages of safety and antioxidant activity, which can improve the quality of frozen products and attract more attention [7]. Some researchers have studied glazing materials such as sodium alginate, green tea extract, and chitosan, which have been shown to extend the shelf life of frozen products [8]. Rosemary is a woody aromatic herb and has been recognized as a food-seasoning product and antioxidant [9]. Rosmarinic acid, as one of the main components of rosemary extract, is a natural, water-soluble phenolic compound, and it is one of the sources of the antioxidant activity of rosemary extract [10]. Li et al. [11] reported the effect of tea polyphenol and rosemary extract combined with chitosan, respectively, on the quality of large yellow croaker refrigerated at 4 ± 1 °C. Their study indicated that dipping with a tea polyphenol and rosemary extract, combined with chitosan glazing, could extend the shelf life of fish by 8–10 days, compared with the control group. Sodium lactate is a low-molecular-weight organic acid sodium salt, which has been used to control microbial growth, improve sensory properties, and extend the shelf life of various food systems [12]. Sallam et al. [13] studied the antimicrobial and antioxidant effects of sodium acetate, sodium lactate, and sodium citrate in refrigerated sliced salmon stored at 1 °C. It was shown that the salmon slices treated by dipping them in 2.5% (*w/v*) aqueous solution of sodium acetate, sodium lactate, or sodium citrate could effectively resist the propagation of various rotten microorganisms, delay lipid oxidation, and prolong the shelf life of the products during cold storage. Bamboo leaf antioxidants have strong antioxidant and antibacterial effects and play an important role in the food industry [14]. Zhang et al. [15] reported the effect of an antioxidant of bamboo leaves combined with different packaging methods on the fresh-keeping of tilapia fillets. The results showed that the tilapia fillets treated with bamboo leaf antioxidants could significantly inhibit the growth and reproduction of microorganisms, reduce the total volatile basic nitrogen (TVB-N) and thiobarbituric acid (TBA) values, slow down the decline rate of the sensory quality, and prolong the shelf life. However, there are few reports on the improvement of tuna quality with such materials. Moreover, the quality changes that occur in tuna glazed with them during frozen storage have not been reported.

In this study, rosmarinic acid, sodium lactate, an antioxidant of bamboo leaf, and a composite of them were applied as glazing materials for frozen tuna. The objective of this study was to evaluate the effect of different glazing materials on physical and chemical changes of frozen tuna during frozen storage.

## 2. Materials and Methods

### 2.1. Materials

A fresh bigeye tuna block was purchased from Dalian Chenyang Science and Technology Development Co., Ltd. It was transported and stored at an ultra-low temperature (−55 °C) after fishing. Rosmarinic acid and an antioxidant of bamboo leaf were purchased as dry powders from Bioengineering (Shanghai) Co., Ltd., and Zhejiang Shengshi Biotechnology Co., Ltd., respectively. The sodium lactate was purchased as a solution from Aladdin Reagent (Shanghai) Co., Ltd.

### 2.2. Preparation of Samples and Glazing Solution 

Tuna were frozen and separated into blocks as soon as they were captured. When the frozen tuna block arrived at the laboratory, several tuna samples were separated right away and analyzed as the initial fresh tuna samples (0 day). The rest of the tuna was randomly divided into six groups: an unglazed group (UG) as the control group; a glazed with water group (WG); a glazed with rosmarinic acid group (RG); a glazed with sodium lactate group (SG); a glazed with an antioxidant of bamboo leaf group (AG); and a glazed with the composite of rosmarinic acid, sodium lactate, and an antioxidant of bamboo leaf group (CG). The mass fractions of RG solution, SG solution, and Ag solution were 0.2%, 3%, and 0.1%, respectively. The RG solution and AG solution were made up by using powder, and the SG was made up by diluting the sodium lactate solution (60%). The CG solution consisted of 96.18% water, 0.3% rosmarinic acid, 3.4% sodium lactate, and 0.12% antioxidant of bamboo leaf [16]. In order to prevent the ice on the tuna layer from cracking, CMC-Na (1%) was added to all the solutions.

### 2.3. Glazing of Tuna and Frozen Storage

Frozen tuna samples were divided into fish blocks (about 450 grams per sample) first, and then the weight was recorded as W_1_. Then, the frozen tuna was dipped into prepared glazing solutions, at 0 °C, for several seconds, and weighed again (record as W_2_). The percentage of glazing was calculated by following Equation (1), and the dipping times of all samples were adjusted to achieve a similar glazing percentage (15% ± 2%).

(1) $$\mathrm{Glazing}\;\mathrm{percentage}\left(\%\right)=\frac{W_{2}-W_{1}}{W_{2}}\times 100\%$$

After glazing, all the samples were packed in polyvinyl chloride bags and stored at −18 °C for 180 days. The samples in each group were taken randomly for analysis every 30 days. Each analysis was repeated at least three times, using different samples.

### 2.4. Water-Holding Capacity (WHC), Drip Loss, and Cooking Loss

The frozen tuna was thawed in air at ambient temperature, and the central temperature changes were measured. The endpoint of thawing was the rise of the central temperature to 5 °C. The surface water of the sample was dried with filter paper. The sample was cut into blocks (about 2 g), and the blocks were weighed as M_1_. Then, the sample blocks were wrapped in filter paper and centrifuged for 10 min (5000 r/min, 4 °C). After centrifuging, the sample blocks were weighed again (M_2_). The water-holding capacity (WHC) was calculated by using Equation (2):

(2) $$\mathrm{WHC}\left(\%\right)=\frac{M_{2}}{M_{1}}\times 100\%$$

Drip loss and cooking loss were determined according to Mousakhani-Ganjeh et al. [17], with some modifications. Before storage, tuna without glazing was weighed and recorded as M_3._ After storage, tuna was thawed and dried of surface water. The weight of tuna was recorded as M_4_. The drip loss was calculated as follows, using Equation (3):

(3) $$\mathrm{Drip}\;\mathrm{loss}\left(\%\right)=\frac{M_{3\;}-M_{4\;}}{M_{3}}\times 100\%$$

The thawed tuna was cut into sample cubes (1 cm × 1 cm × 1 cm) and weighed as M_5_. Then, tuna was placed in resealable plastic bags and cooked in a water bath at 85 °C for 10 min. After cooling, tuna sample cubes were taken out of the bag and reweighed as M_6_. The cooking loss was calculated as follows, using Equation (4):

(4) $$\mathrm{Cooking}\;\mathrm{loss}\left(\%\right)=\frac{M_{5\;}-M_{6}}{M_{5}}\times 100\%$$

### 2.5. Low-Field Nuclear Magnetic Resonance (LF-NMR) Analysis

A low magnetic field Benchtop Pulsed NMR analyzer (Niumag) was used for measurements of transversal (T_2_) relaxation times in this study. Tuna samples were divided into blocks (2 cm × 2 cm × 2 cm) and wrapped in plastic wrap. The samples were pushed into the NMR analyzer in order, and the T_2_ measurement parameters were set as follows: sampling frequency SW = 100 kHZ, repeated sampling time 4000 ms, echo time TE = 0.500, and echo number NECH = 8000. According to the CPMG exponential attenuation curve generated by the analysis software, the transverse relaxation time, T_2_, image was obtained by iterative inversion.

### 2.6. Determination of Protein Degradation and Fat Oxidation

#### 2.6.1. Preparation of the Myofibrillar Protein

Myofibrillar protein was prepared according to the method of Niu [18]. After thawing, samples were minced and mixed with PBS solution (20 mL). They were then homogenized (6000 rpm, 3 min) and centrifuged (10,000 g, 4 °C, 20 min). Following this, the obtained sediments were washed with NaCl solution (12 mL, 3%) three times, to extract more water-soluble substances. The supernatant was collected, and the myofibrillar protein concentration was determined by the Biuret assay method.

#### 2.6.2. Determination of the Total Volatile Basic Nitrogen (TVB-N)

The TVB-N was determined according to the method in the GB 5009.228-2016 standard [19].

#### 2.6.3. Determination of malondialdehyde (MDA) 

The ground tuna samples (1 g) were homogenized in normal saline (9 mL) and centrifuged for 10 min (2500 rpm, 4 °C. After centrifuging, the supernatant was separated and determined by visible spectrophotometry, with a malondialdehyde kit (Jiancheng Bioengineering Institute, Nanjing, China). All analyses were performed in triplicate for each sample.

### 2.7. Color and Texture

The surface color of samples was determined by a colorimeter (CR-400, Konica Minolta Corporation, Tokyo, Japan). The colorimeter was calibrated with a white standard plate (Y = 88.5, x = 0.3149, and y = 0.3222) provided by the manufacturer. The a* value represents the degree of redness to greenness, and it was used as the basis for judging the color change. The positive and negative sides of each sample were determined twice. Each group of samples was measured three times, in parallel. 

Each group of tuna samples was cut into six blocks (1 cm × 1 cm × 2 cm), and the hardness was determined by a texture analyzer (TA.XT Plus texture analyzer, Precisa International (Shanghai) Co., Ltd). All samples were compressed twice, and the average value was taken as the result. The texture was assessed, using a texture analyzer, and the parameters were set as follows: speed before test = 3 mm/s; test speed = 1 mm/s; speed after test = 1 mm/s; compression degree = 50%; dwell interval = 5 s; probe type = auto-5 g; data collection rate = 200; and ambient temperature = 11–16 °C.

### 2.8. Free Amino Acid (FAA)

Refer to Yu [20] et al. for the determination method. A Hitachi L-8800 automatic amino acid analyzer was used for analysis.

### 2.9. Statistical Analysis

Experimental data was statistically analyzed, using SPSS 21.0 (IBM, Chicago, Illinois, USA), and the results are reported as the mean ± standard deviation. Analysis of variance (ANOVA) was carried out to determine differences, and *p* < 0.05 was considered statistically significant. All the curves in this paper were drawn by origin Pro 2016 (OriginLab, Northampton, MA, USA).

## 3. Results and Discussion

### 3.1. Physicochemical Analysis

#### 3.1.1. Changes in the Water-Holding Capacity, Drip Loss, and Cooking Loss

The water-holding capacity (WHC), drip loss, and cooking loss are primary determinants of sensory appeal. Additionally, they are important indicators used to measure tuna freshness, which is related to tuna muscle protein and its organizational structure [21].

The cooking loss of all groups is shown in Table 1. The cooking loss of UG was significantly higher than that of other groups. This may have been due to the denaturation of tuna myofibrillar protein during the frozen storage, which leads to an increased loss of nutrients and water in cooking [22]. In the process of storage, with the extension of the storage time, the overall cooking loss did not change significantly, and the CG, RG, and SG had a low average cooking loss among all the groups. This illustrated that the cooking loss was less affected by the storage time and more affected by the glazing materials. Using rosemary acid, sodium lactate, and a bamboo leaf antioxidant as the glazing material could keep the myofibrillar protein stable, maintain the muscle structure, and reduce the cooking loss. 

**Figure 1:**
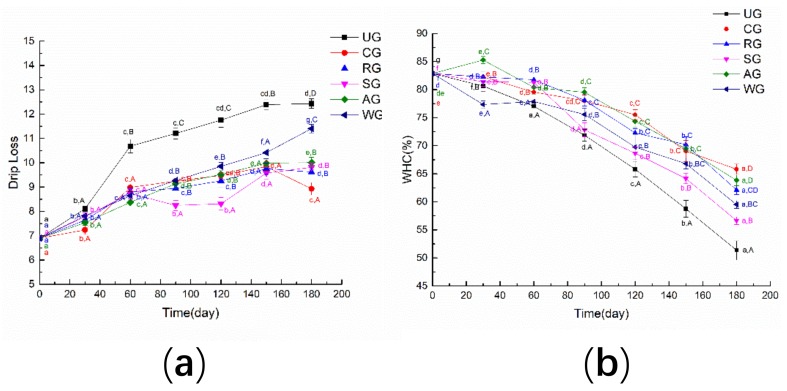
The drip loss (**a**) and water-holding capacity (WHC) (**b**) of tuna glazed with different materials. Different lowercase letters in the same treatment indicate significant differences (*p* < 0.05) between different time points. Different capital letters at the same time point indicate significant differences (*p* < 0.05) between different treatments. Note: UG: an unglazed group; CG: a glazed with the composite of rosmarinic acid, sodium lactate, and an antioxidant of bamboo leaf group; RG: a glazed with rosmarinic acid group; SG: a glazed with sodium lactate group; AG: a glazed with an antioxidant of bamboo leaf group; WG: a glazed with water group.

Figure 1a shows the changes in drip loss of tuna with different glazing materials during frozen storage. It can be seen that the drip loss of unglazed tuna samples increased the fastest and the drip loss of WG was also increased during the storage time. The drip loss of AG, SG, RG, and CG growth tended to continuously increase with storage, and the CG tuna had the lowest drip loss after 180 days of storage. As shown in Figure 1b, the water-holding capacity of all samples exhibited decreasing trends during storage. After six months, the water-holding capacity of UG, RG, SG, AG, WG, and CG tuna samples decreased to 51.36%, 62.1%, 56.65%, 63.82%, 59.5%, and 65.81%, respectively.

At the beginning of the 30 days, there was no significant difference in the drip loss of each group. After 90 days of storage, the drip loss of UG and WG was significantly higher than that of other groups. It was noted that the drip loss of CG, RG, and AG was significantly lower, while the water-holding capacity (WHC) of CG, AG, and RG was significantly higher. This may have been due to the glazing treatment, especially CG, which can prevent the destruction of hydrophobic and hydrophilic bonds around proteins during freezing storage [23]. 

#### 3.1.2. Changes in the Color of Samples

**Figure 2:**
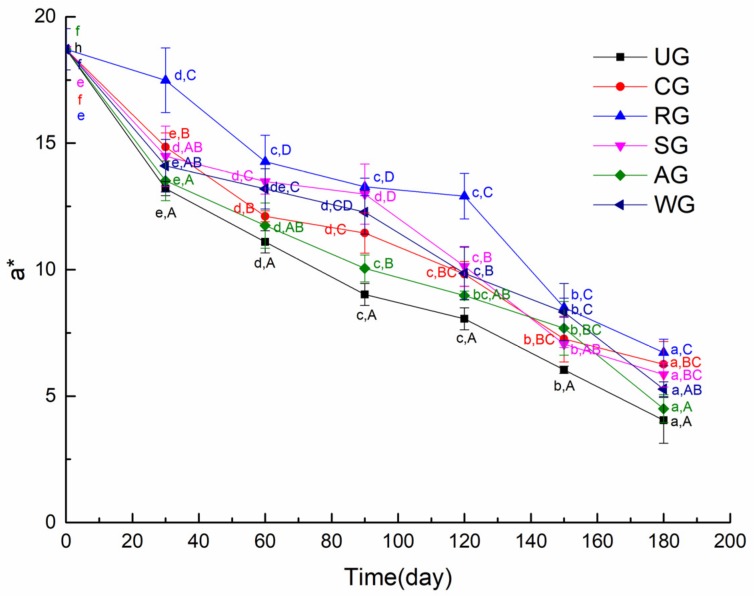
The color of tuna glazed with different materials. Different lowercase letters in the same treatment indicate significant differences (*p* < 0.05) between different time points. Different capital letters at the same time point indicate significant differences (*p* < 0.05) between different treatments.

Tuna muscle’s color is an important factor for the consumer in determining whether or not to accept the tuna [24]. Fresh tuna’s bright red color is primarily due to the presence of relatively large amounts of the ferrous myoglobin oxygen complex, and its color will gradually turn brown–red because of degradation of the derivative [25]. The color of muscle is mainly determined by myoglobin, which has three redox forms: deoxymyoglobin, oxymyoglobin, and ferrimyoglobin. The oxidation of myoglobin will lead to dull-colored tuna meat [26]. In this paper, the color of the muscle is characterized by a* value. As shown in Figure 2, the initial a* values, which reflect the muscle color of samples, were close to 19. The a* values of CG, SG, and WG were not significantly different after six months of frozen storage. RG had significantly higher redness scores compared to the other groups at the end of storage. This is because rosmarinic acid is a strong antioxidant. Djenane et al. [27] found that the a* parameter of the mixed treatment group of rosemary and VC could still reach the value of 29 days, which was equivalent to that of the group without antioxidants for 12 days. The tuna with AG had a poor color, which was caused by the brown antioxidant of bamboo (AOB) leaf residues on the surface of the tuna.

#### 3.1.3. Texture Analysis

**Figure 3:**
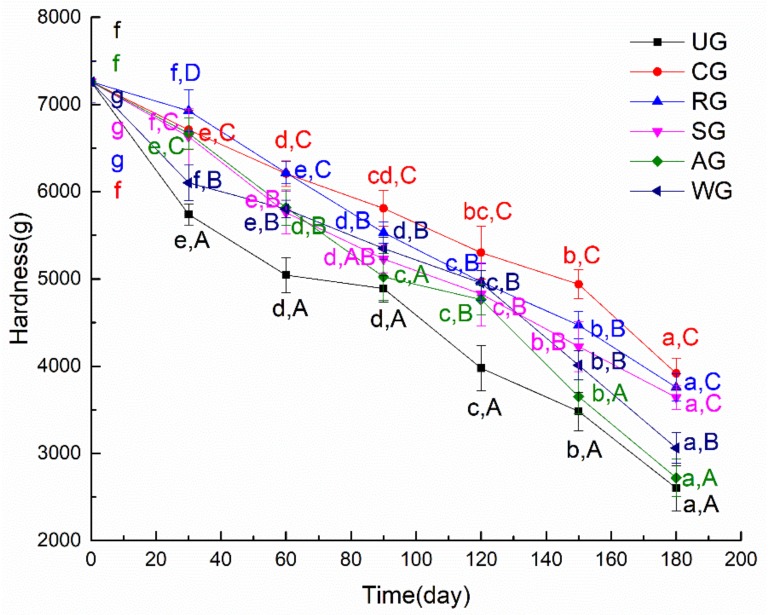
The hardness of tuna glazed with different materials. Different lowercase letters in the same treatment indicate significant differences (*p* < 0.05) between different time points. Different capital letters at the same time point indicate significant differences (*p* < 0.05) between different treatments.

The texture is another important quality parameter for tuna and includes hardness, resilience, chewiness, adhesion, and so on [28]. The denaturation of protein and the decomposition of microorganisms in tuna meat will lead to a decrease of texture. In this study, the texture of the tuna samples was quantified by measuring the hardness. Figure 3 shows the hardness changes of tuna glazed with different materials during 180 days. It illustrates that the hardness of all groups showed a downward trend. There are two reasons for this phenomenon: on the one hand, in the process of frozen storage, the volume of free water in the tuna increased and expanded while it was frozen into ice, which led to the deformation of myofibrillar cells and the hardness of fish meat decreased after thawing; on the other hand, the hardness of muscle is affected by the content of salt-soluble protein in muscle, where the higher the degree of protein denaturation, the faster the hardness decreases [29,30]. Hardness significantly decreased in all groups after 180 days of frozen storage, but the hardness of RG, CG, and SG was significantly higher than that of other groups. This illustrates that the texture of tuna was greatly affected by the glazing material. CG had the highest hardness after 180 days of frozen storage. This illustrated that CG could reduce the denaturation degree of protein; maintain the muscle structure, which could slow down the decrease of hardness during frozen storage; and maintain the quality of tuna.

### 3.2. Low-Field Nuclear Magnetic Resonance (LF-NMR) Relaxation Time (T_2_) and Moisture Distribution

LF-NMR is used to determine the content of water in the sample by measuring the attenuation of the ^1^H proton transverse relaxation time (T_2_) in tuna samples [31].

**Figure 4:**
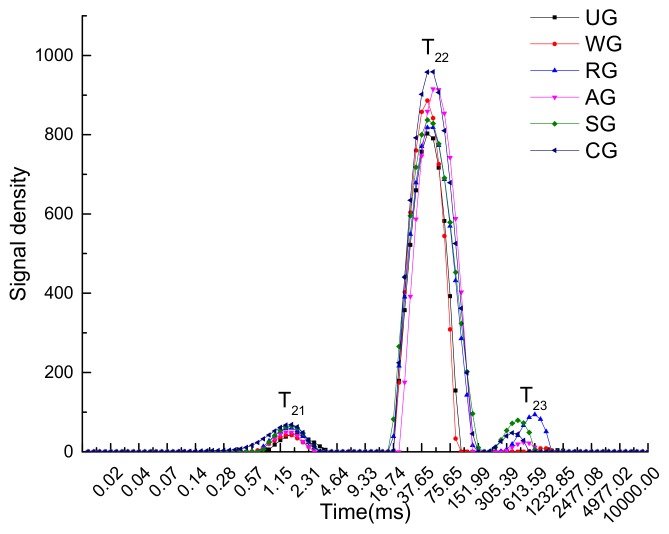
Transverse relaxation time of tuna glazed with different materials.

Figure 4 shows the T_2_ transverse relaxation times of the tuna samples after six months of storage. The distribution shows that the water types in tuna fish muscle can be divided into three forms: bound water (T_21_), which is tightly associated with macromolecules that have a relaxation time centered at 0–10 ms; immobilized water (T_22_), which is located in the myofibrillar network and has a relaxation time centered at 10–150 ms; and free water (T_23_), which has a relaxation time of more than 150 ms. The transverse relaxation time (T_2_) reflects the bonding force between water and muscle tissue, and the peak area of the curve represents the water component [32]. It was found that the moisture content of CG was the highest, while the moisture contents of UG and WG were both lower. The distribution result of tuna samples illustrated that the immobilized water (T_22_) of CG and AG was higher. This may have been due to the fact that CG and AG have significantly lower drip loss and higher water-holding capacity. This indicates that increasing the water-holding capacity and reducing the thawing loss are helpful for increasing the water content. There is a certain correlation between the water-holding capacity and muscle water content. While the drip loss and cooking loss are large, the free water will be greatly lost, and the immobilized water will be converted into free water in large quantities [33]. 

Table 2 shows the changes of T_21_, T_22_, and T_23_ in tuna samples with different treatments during frozen storage, which are expressed as the proportion of bound water (P_21_), the proportion of immobilized water (P_22_), and the proportion of free water (P_23_), respectively. Immobilized water is the main component in tuna samples, with P_22_ accounting for more than 90% of samples, while bound water and free water are secondary water components. Table 2 illustrates that the moisture content of all samples showed a downward trend. The increase of P_22_ in the first 120 days may have been caused by the loss of free water. Subsequently, P_22_ began to decrease due to a decrease of the water-holding capacity and drip loss of the myofibrillar network in the process of frozen storage. Shi et al. [34] studied the effect of glazing and rosemary extract on the preservation of mud shrimp during frozen storage and presented a similar conclusion. The proportion of free water of all shrimp samples decreased during frozen storage, while the immobilized water percentage increased during the first eight weeks of storage and then decreased slightly. After 180 days of storage, the P_23_ of UG was the lowest, and its water loss was the most serious. The P_22_ of RG decreased the fastest, which indicates that there was a decreased bonding force of water in RG tissue, leading to the conversion of immobilized water to free water. 

**Figure 5:**
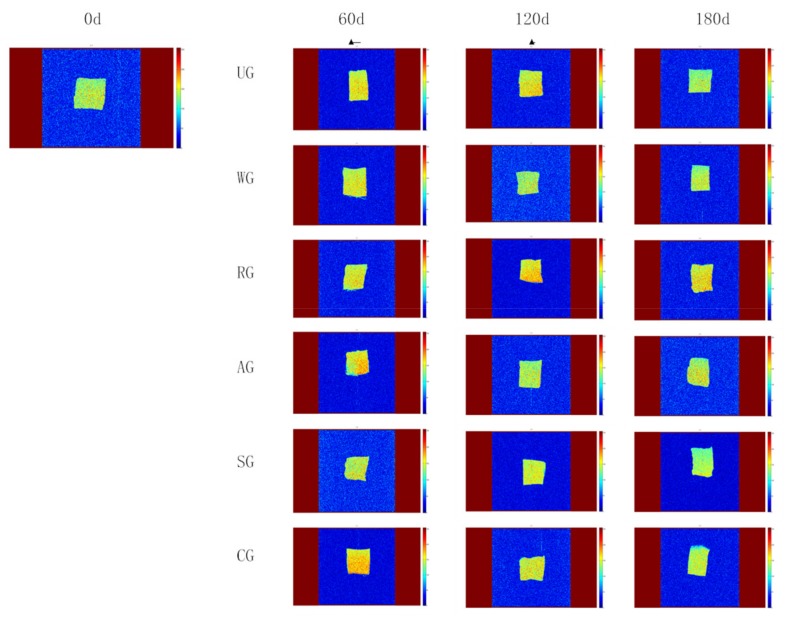
False-color image of the water proton density in tuna glazed with different materials. d, day.

Figure 5 shows the distribution of water detected by using LF-NMR technology, where the signal intensity reflects the moisture content in the samples; the brighter the color in the figure, the higher the moisture content of the sample [35]. The picture of fresh tuna samples shows yellow and red, indicating that the signal intensity is high and the moisture content is the highest. During frozen storage, UG showed a green color, which indicated that the water content of the UG group decreased rapidly, and the color of RG, SG, and AG was bright locally, indicating that the distribution of moisture content was uneven. After 180 days of storage, CG had an intensive and even signal intensity among all the groups, which shows that CG has the best water retention effect and can effectively reduce water loss.

### 3.3. Protein Degradation 

#### 3.3.1. Changes in the Myofibrillar Protein Content

**Figure 6:**
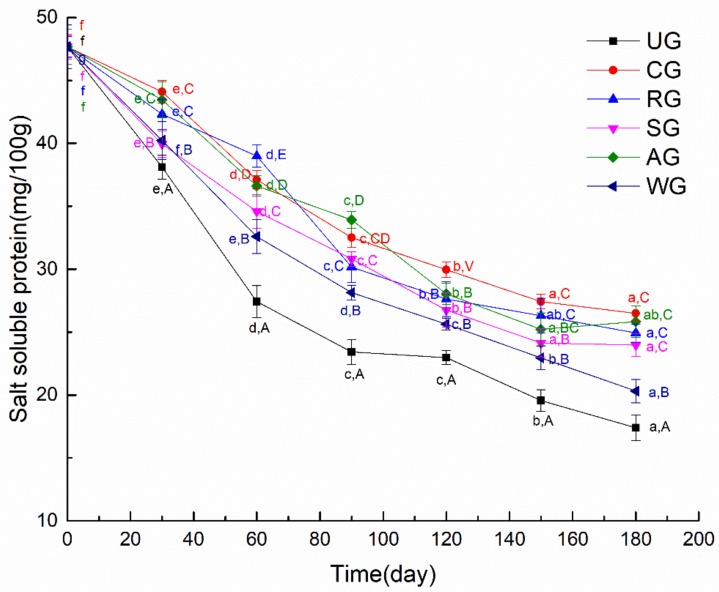
The myofibrillar protein content of tuna glazed with different materials. Different lowercase letters in the same treatment indicate significant differences (*p* < 0.05) between different time points. Different capital letters at the same time point indicate significant differences (*p* < 0.05) between different treatments.

Changes in the myofibrillar protein content from glazed tuna are shown in Figure 6. The total myofibrillar protein content of all tuna samples decreased gradually with the prolonged frozen storage time. The myofibrillar protein content of UG tuna samples decreased the fastest in all groups. This illustrated that the myofibrillar protein of UG tuna samples was seriously oxidized due to direct contact with air. This phenomenon may have occurred because the disulfide bond formed by sulfhydryl oxidation results in a decrease of the salt-soluble protein content [36]. There was no significant difference in the myofibrillar protein content of tuna samples with SG and AG during frozen storage, while tuna samples with WG exhibited a significantly lower value than that of the other three groups of tuna samples. These results suggested that glazed tuna with rosmarinic acid and an antioxidant of bamboo leaf can slow down myofibrillar protein content changes and protect the protein from oxidation compared to UG, and CG had the highest myofibrillar protein content among all groups. The RG had the lower content of salt-soluble protein compared to CG, which may have been due to some denaturation of proteins [37].

#### 3.3.2. Determination of Total Volatile Basic Nitrogen (TVB-N)

**Figure 7:**
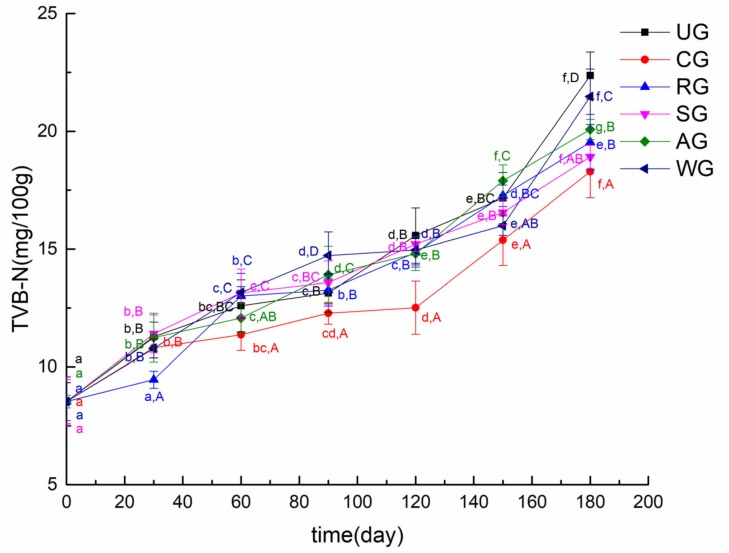
The total volatile basic nitrogen (TVB-N) value of tuna glazed with different materials. Different lowercase letters in the same treatment indicate significant differences (*p* < 0.05) between different time points. Different capital letters at the same time point indicate significant differences (*p* < 0.05) between different treatments.

TVB-N is an important indicator used to measure the quality of seafood. It increases with deteriorative changes of seafood [38]. The variations in TVB-N of the tuna samples are presented in Figure 7. The initial TVB-N value of the tuna sample was 8.5 mg/100 g muscle in this study. Seafood is considered not fresh when its TVB-N value exceeds 30.0 mg/100 g [39]. The TVB-N value of all groups increased slowly in the first 30 days, but displayed faster growth after 120 days. The increase of the TVB-N value can be attributed to ammonia produced by the endogenous enzymes and spoilage microorganisms, which substantially increase with the storage time [40]. In the early stage of storage, the activity of endogenous enzymes in fish was low, and the number of microorganisms was relatively small. With the increase of endogenous enzyme activity and the number of microorganisms, the value of TVB-N increased rapidly in the late stage of storage [41]. After 180 days of storage, the TVB-N value of UG reached the value of 22.37 mg/100 g muscle, while the TVB-N value of CG was close to 18.29 mg/100 g muscle. Ozogul et al. [42] studied the effect of different concentrations of rosemary extract on the quality of vacuum-packaged sardines. They illustrated that rosemary extract significantly inhibited the production of TVB-N in sardines. In this paper, the difference in the TVB-N values of CG, SG, and AG was not significant, but those values were significantly higher than that of WG. It was obvious that the TVB-N value of CG increased more slowly than others. It could be concluded that glazing with the composite of rosmarinic acid, sodium lactate, and bamboo leaf antioxidant effectively slowed down the growth of microorganisms and inhibited the activity of endogenous enzymes.

### 3.4. Lipid Oxidation 

#### 3.4.1. Changes in Malondialdehyde (MDA)

**Figure 8:**
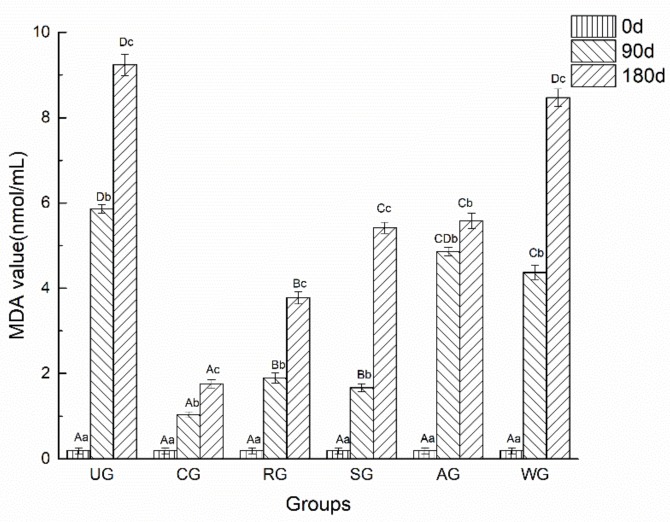
The malondialdehyde (MDA) value of tuna glazed with different materials. Different lowercase letters in the same treatment indicate significant differences (*p* < 0.05) between different time points. Different capital letters at the same time point indicate significant differences (*p* < 0.05) between different treatments. d, day.

Lipid oxidation in tuna was quantified by determining the content of MDA, which is the final product of lipid oxidation. Figure 8 shows the MDA values of all tuna groups at 0, 90, and 180 days, respectively. It was observed that the MDA values of UG increased rapidly with the storage time and peaked at 180 days of frozen storage. This may have been due to the accelerated lipid oxidation of the UG in direct contact with air. There were no significant differences among the groups at the initial 30 days; however, significant differences were observed at day 90 between samples of CG, RG, and SG and those of the rest of the treatments. It was illustrated that the lipid oxidation of tuna glazed with rosmarinic acid and sodium lactate could be inhibited in a short time. Seydim et al. [43] also found that adding rosemary extract can effectively inhibit the fat oxidation of ostrich meat cake under vacuum packaging. This may happen because rosmarinic acid, as the main component of rosemary, can over-block the free radical reaction and effectively inhibit lipid peroxidation [44]. In the next 90 days, the MDA value of AG increased the slowest. This may have been because AOB is rich in flavonoids, lactones, and phenolic acids, which can block the chain reaction of the automatic oxidation of fat chains and scavenge a variety of active oxygen radicals. Because of the synergistic effect of rosmarinic acid, sodium lactate, and AOB, the MDA values of CG had the lowest MDA value after 180 days of storage. 

#### 3.4.2. Changes in Free Amino Acid (FAA)

Amino acids have a unique flavor. The amino acid content can interact with other components, thus affecting the flavor of fish during storage [45]. Table 3 shows the FAA changes of all tuna samples after 180 days of frozen storage. The FAA content of UG was significantly higher than that of other groups, indicating that the protein extensive degradation degree of UG was higher, and the tuna samples were seriously deteriorated. After the frozen storage, the FAA content of RG was the lowest, followed by AG and CG. This could have been due to the strong antioxidant properties of rosemary and bamboo leaf, which inhibited degradation of the protein. Shi et al. [34] used rosemary extract as glazing material to glaze mud shrimp, slowing down the change of sulfhydryl content and protecting the protein from oxidation. Some amino acids are called peculiar-smell amino acids, especially serine, glutamic acid, glycine, and histidine. For example, histidine will cause certain bitterness in fish meat, and arginine will increase the freshness of fish meat [46]. SG and UG tuna have more histidine and arginine, which will cause more bitterness and a greater taste decline. CG, RG, and AG have a low content of peculiar smell amino acids, which shows that they can maintain the flavor of tuna to the greatest extent. 

## 4. Conclusions

This study has demonstrated the effectiveness of rosmarinic acid, antioxidants of bamboo leaves, and sodium lactate as glazing materials for frozen tuna. They can significantly improve the fresh-keeping effect of tuna and the draught of protein. The water-holding capacity of all samples showed a significant decrease during frozen storage. The cooking loss was less affected by the frozen storage time, and CG, RG, and SG exhibited lower cooking loss. The hardness and a* value of RG, SG, and CG decreased slowly, indicating that sodium lactate and rosmarinic acid were effective for the preservation of the texture and color of tuna. Antioxidants of bamboo leaf had a strong inhibition effect on MDA from 90 to 180 days, but had a great influence on the color of tuna. As shown from the results of NMR, CG has a bright color and uniform distribution among all groups, and it was illustrated that CG has the best water retention for tuna. After 180 days of storage, CG samples had the highest protein content and water-holding capacity, the lowest TVB-N value and MDA value, and a low FAA content, which can maximize the freshness of tuna. Therefore, CG can be considered the most suitable material for tuna ice coatings.

## Figures and Tables

**Table 1:** The cooking loss(%) of tuna glazed with different materials.

Treatment	Storage Time (day)					
	30	60	90	120	150	180
UG	31.09 ± 2.6 Ec	29.71 ±0.4 Eb	27.64 ± 2.4 Ca	29.86 ± 2.4 Cb	27.54 ± 1.9 Da	33.02 ± 1.4 Dd
CG	23.69 ± 1.2 Ba	26.63 ± 1.5 Ccd	27.70 ± 2 Cd	24.42 ± 2.1 Bab	24.57 ± 2.2 Cab	26.02 ± 1.6 Cc
RG	18.50 ± 1.5 Aa	28.51 ± 0.6 Dd	30.42 ± 1.7 De	24.58 ± 0.6 Bb	18.30 ± 1.8 Aa	25.63 ± 1.0 Cc
SG	30.21 ± 2.1 Dd	23.72 ± 1.7 Ab	27.88 ± 1.7 Cc	20.39 ± 1.8 Aa	21.67 ± 1.2 Bab	19.88 ± 1.4 Aa
AG	30.29 ± 1.3 Dd	24.48 ± 1.8 ABab	23.91 ± 1.6 Ba	29.82 ± 1.9 Cd	27.14 ± 0.5 Dc	24.97 ± 1.3B Cab
WG	28.20 ± 2.6 Ccd	25.40 ± 1.8 Bb	21.27 ± 0.7 Aa	29.95 ± 2.1 Cd	27.76 ± 0.7 Dc	23.83 ± 0.5 Bab

Different lowercase letters in the same treatment indicate significant differences (*p* < 0.05) between different time points. Different capital letters at the same time point indicate significant differences (*p* < 0.05) between different treatments. Note: UG: an unglazed group; CG: a glazed with the composite of rosmarinic acid, sodium lactate, and an antioxidant of bamboo leaf group; RG: a glazed with rosmarinic acid group; SG: a glazed with sodium lactate group; AG: a glazed with an antioxidant of bamboo leaf group; WG: a glazed with water group.

**Table 2:** The peak area proportion of tuna glazed with different materials.

The proportion		UG	WG	RG	AG	SG	CG
0 day	T2 peak area	13,000.83781					
	T21 peak area proportion	0.04					
	T22 peak area proportion	0.95					
	T23 peak area proportion	0.01					
60 days	T2 peak area proportion	10,622.29	9010.84	9186.84	8788.45	9384.28	11,389.84
	T21 peak area proportion	0.04	0.05	0.03	0.04	0.03	0.04
	T22 peak area proportion	0.96	0.91	0.96	0.89	0.96	0.96
	T23 peak area proportion	0.00	0.04	0.01	0.08	0.01	0.01
120 days	T2 peak area proportion	9260.24	7854.69	8522.56	8616.11	8871.52	9574.82
	T21 peak area proportion	0.04	0.01	0.04	0.04	0.03	0.04
	T22 peak area proportion	0.96	0.99	0.96	0.96	0.97	0.96
	T23 peak area proportion	0.01	0.00	0.00	0.01	0.00	0.00
180 days	T2 peak area proportion	6218.72	6421.40	8018.95	7758.65	8473.01	9208.06
	T21 peak area proportion	0.05	0.04	0.05	0.04	0.05	0.06
	T22 peak area proportion	0.95	0.96	0.90	0.95	0.91	0.92
	T23 peak area proportion	0.00	0.00	0.06	0.01	0.04	0.02

**Table 3:** The free amino acid (FAA) value of tuna glazed with different materials after 180 days of storage.

Type	WG	RG	AG	SG	CG	UG
Asp	0.49 ± 0.13 AB	0.39 ± 0.06 A	0.62 ± 0.04 C	0.34 ± 0.02 A	0.54 ± 0.15 B	0.37 ± 0.03 A
Thr	1.82 ± 0.07 A	1.88 ± 1.01 A	1.88 ± 0.62 A	2.82 ± 0.44 B	3.12 ± 0.49 B	2.83 ± 0.31 B
Ser	2.04 ± 0.24 AB	1.63 ± 0.33 A	1.73 ± 0.21 A	2.46 ± 0.20 B	3.18 ± 0.08 C	2.40 ± 0.07 B
Glu	0.80 ± 0.09 A	1.50 ± 0.15 BC	1.80 ± 0.09 C	1.27 ± 0.06 B	0.90 ± 0.04 A	0.78 ± 0.17 A
Gly	3.28 ± 0.36 B	2.72 ± 0.29 A	2.23 ± 0.17 A	5.19 ± 0.46 C	3.27 ± 0.30 B	5.81 ± 0.26 C
Ala	8.05 ± 0.54 B	4.93 ± 0.95 A	10.73 ± 1.91 C	10.74 ± 0.95 C	15.58 ± 4.37 D	9.61 ± 1.01 BC
Val	5.13 ± 0.55 C	1.64 ± 0.27 A	4.05 ± 0.62 B	4.05 ± 1.89 B	4.79 ± 1.44 BC	4.11 ± 0.46 B
Met	2.91 ± 0.09 D	1.48 ± 0.34 A	2.50 ± 1.02 C	1.31 ± 0.09 A	2.10 ± 0.36 BC	1.80 ± 0.06 B
Ile	2.58 ± 0.62 C	2.05 ± 1.32 A	2.09 ± 1.17 A	2.26 ± 0.56 B	3.06 ± 1.05 D	2.22 ± 0.63 B
Leu	4.49 ± 1.15 C	3.30 ± 0.84 A	3.69 ± 1.19 B	3.47 ± 1.66 AB	5.16 ± 1.99 D	3.42 ± 0.47 A
Tyr	3.92 ± 1.17 C	1.67 ± 0.71 A	3.50 ± 0.20 C	2.65 ± 0.53 B	2.76 ± 0.96 B	2.62 ± 0.73 B
Phe	3.04 ± 1.37 C	1.51 ± 0.43 A	2.13 ± 0.69 B	2.05 ± 0.36 B	2.20 ± 1.36 B	2.16 ± 0.64 B
Lys	65.37 ± 6.18 C	38.05 ± 3.18 A	57.90 ± 3.11 B	65.85 ± 3.81 C	60.54 ± 5.47 BC	70.90 ± 4.42 D
His	290.10 ± 12.16 D	203.59 ± 14.49 A	231.11 ± 6.63 B	334.78 ± 18.42 E	270.60 ± 9.38 C	359.45 ± 22.23 F
Arg	0.42 ± 0.13 A	1.02 ± 0.10 C	0.70 ± 0.34 B	1.30 ± 0.23 CD	0.52 ± 0.36 AB	1.51 ± 0.67 D
Pro	1.12 ± 0.29 A	1.01 ± 0.60 A	1.92 ± 0.78 B	1.31 ± 0.35 A	2.55 ± 1.05 C	1.14 ± 0.27 A
Total	395.56 ± 5.20	268.36 ± 7.05	328.57 ± 13.72	441.85 ± 20.50	380.87 ± 21.57	471.13 ± 19.7

Different capital letters for the same type indicate significant differences (*p* < 0.05) between different treatments. Note: Asp: Asparticacid; Thr: Threonine; Ser: Serine; Glu: Glutamicacid; Gly: Glycine; Ala: Alanine; Val: Valine; Met: Methionine; Ile: Isoleucine; Leu: Leucine; Tyr: Tyrosine; Phe: Phenylalanine; Lys: Lysine; His: Histidine; Arg: Arginine; Pro: Proline;.
